# How Do Consumers Understand Food Processing? A Study on the Brazilian Population

**DOI:** 10.3390/foods11162396

**Published:** 2022-08-10

**Authors:** Jordanna Santos Monteiro, Eduardo Yoshio Nakano, Renata Puppin Zandonadi, Raquel Braz Assunção Botelho, Wilma Maria Coelho Araujo

**Affiliations:** 1 Department of Nutrition, School of Health Sciences, University of Brasilia (UnB), Campus Darcy Ribeiro, Asa Norte, Brasilia 70910-900, DF, Brazil; 2 Department of Statistics, Central Institute of Sciences, University of Brasilia (UnB), Campus Darcy Ribeiro, Asa Norte, Brasilia 70910-900, DF, Brazil

**Keywords:** food classification, food-based dietary guidelines, consumer, understanding, processed food

## Abstract

Food guides are official documents that guide consumers’ food choices. They inform the qualitative classification of food groups and messages on how to adopt a healthy diet. The classifications and nomenclatures adopted in these documents vary according to cultural, nutritional, and scientific criteria. This study aimed to evaluate the understanding of Brazilian consumers regarding food classification according to the *Food Guide for the Brazilian Population* (FGBP) concepts. An instrument was constructed to assess consumer understanding. It was named “Understanding of the Level of Processing of Food” (ULPF) and validated according to the concepts of constructs presented by psychometrics such as the Delphi methodology. The instrument was composed of 36 items approved by experts (concordance > 80% and with good internal consistency). A total of 2333 Brazilians from all regions participated in the study. The results suggest that food classification according to the level of processing was difficult for participants to understand. About 85% of them did not understand or did not know the definitions and classification of food and preparations according to food and science technology (FST) and the FGBP. More than 50% of the participants believed that it was easier to classify food according to food groups.

## 1. Introduction

Food-based dietary guidelines (FBDG) are official documents written in easy-to-understand language that intend to influence a population’s eating behavior based on national food, nutrition, and health policies and programs [1,2,3,4,5,6]. In constructing FBDG, cultural and health standards and government recommendations must be considered, as well as dietary patterns and food available for consumption in the country [1].

Food-based dietary guidelines (FBDG) should be practical, accessible, and with options for different population groups. Since FBDG are food and nutrition education instruments for the population, they must describe food with appropriate classification and nomenclature specific to each country. According to the Food and Agriculture Organization of the United Nations (FAO), the classification of food used in food guides should be based on food groups and nutrient sources. Food classification by food groups categorizes foods according to their origin, nutritional properties, marketing characteristics, or jointly [4]. Therefore, FBDG must consider the most appropriate classification of food from a scientific point of view and “translate” this information for the consumer, avoiding inconsistencies and ambiguous information, allowing understanding and knowledge of the population [1,2,3,4,5,6] so FBDG will achieve their primary goal to guide consumption of food and food groups, and dietary patterns, that optimize the intake of nutrients to promote health and prevent chronic diseases in the population. People must know and understand the FBDG content to achieve this primary goal.

In Brazil, studies carried out on the access to and knowledge of the Food Guide for the Brazilian Population show that few individuals read it, that Brazilians have difficulty understanding the messages within the guide, and that their information and knowledge are linked only to the nutritionist profession [7,8,9]. It is noteworthy that there is a difference between knowledge and understanding. Not everyone who knows can understand because understanding is associated with the ability to judge, give an opinion about something and understand [10,11]. There are no studies that assess the understanding of the Brazilian Food Guide or the understanding of food classifications used in it. Therefore, this study aimed to (i) evaluate the understanding of Brazilian consumers regarding the classification of food according to the concepts of the Food Guide for the Brazilian Population (FGBP); (ii) verify if the consumer better understands the classification according to the concept of food science and technology (FST).

## 2. Materials and Methods

### 2.1. Study Design

This qualitative−quantitative, cross-sectional study was performed with Brazilian consumers. The first step was to develop and validate the instrument “Understanding of the Level of Processing of Food” (ULPF) to achieve this study goal. The instrument validation occurred according to Boateng et al. [12] and the Delphi method [13,14,15]. The University of Brasília/Brazil Ethics Committee approved this project (CAAE 38084620.1.0000.8093). After validation, the ULPF was sent electronically to consumers in all of Brazil’s geographic regions.

### 2.2. Construction of the Instrument “Understanding of the Level of Processing of Food” (ULPF)

Considering the definitions of the items and the domains, the initial instrument presented 106 items. The items proposed for the instrument were described according to the nomenclature, the food classification (food groups and processing level) described in the 89 food-based dietary guidelines available on the FAO website (http://www.fao.org/nutrition/education/fooddietary-guidelines/home/en/, (accessed on 12 January 2020) [6] and the Brazilian legislation [16,17,18,19,20,21].

**Figure 1:**
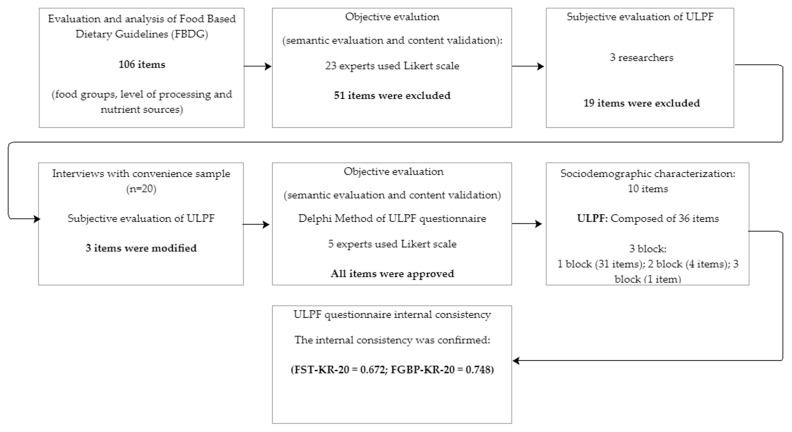
Stages of the construction, content validation, and semantic evaluation of “Understanding of the Level of Processing of Food” (ULPF).

To assess the items’ clarity and relevance, 59 experts (university professors, food technologists, food scientists, and nutritionists) were invited, and 23 agreed, to participate in the instrument evaluation. Item clarity was assessed using a 5-point scale: 1 (I did not understand at all), 2 (I understood a little), 3 (I understood almost everything, with reservations), 4 (I understood almost everything), and 5 (I completely understood). Relevance was assessed using a 5-point scale: 1 (inadequate), 2 (very little adequate), 3 (little adequate), 4 (adequate) and 5 (very adequate). If necessary, they were also asked to include their suggestions for item modification. Data were analyzed considering each evaluated item and the suggestions presented for the items’ reformulation. The criterion for keeping the item in the instrument was to obtain at least 80% agreement among the experts on each item. Items that did not reach 80% were reworked and re-evaluated. When recommended by experts, the item was excluded, according to the Delphi methodology [13,14,15]. At the end of this phase, the experts recommended that the instrument should only have items related to the food processing level because of the objective of the study (Figure 1).

The final version of this first step included the constructive and operational definitions of the Food Guide for the Brazilian Population (FGBP) and the definitions of food processing available in the food science and technology (FST) literature, integrating the following definitions: *in natura* food, minimally processed food, processed food and ultra-processed food. After the recommendation of the 23 experts, three university researchers in food science and technology and nutrition (W.M.C.A.; R.P.Z.; R.B.A.B.) evaluated the items before they were sent out for evaluation interviews [12,22,23,24]. The interviews were composed of a convenience sample of 20 respondents, aged between 18 and 70 years, from different social classes and educational levels to assess their understanding of the items. The interviews were conducted individually, and when in a group, the maximum number of participants was three. Item clarity was assessed by interviews using a 5-point scale: 1 (I did not understand at all); 2 (I understood a little); 3 (I understood almost everything, with reservations); 4 (I understood almost everything); and 5 (I completely understood). If necessary, they were also asked to include their suggestions for item modification. After all the interviews, the suggestions were accepted for modifying the items that were not clear or that were too scientifically described. The examiner read the items aloud, and each participant was invited to judge them based on their comprehension and to suggest a better description for the item when relevant. Then, the instrument was rewritten and sent to a new analysis stage by the experts. At this step, only five of the 23 experts accepted to participate. Thus, the ULPF was again submitted to content analysis and semantic analysis.

After experts’ approval, the ULPF was spread nationwide using a convenience sample by the snowball method [12]. The instrument was applied through the Google Forms™ platform to a convenience sample of Brazilian adults (≥19 y/o) from all Brazilian regions. Participants were recruited through social media advertising (Facebook™, Instagram™, and WhatsApp™). The data collection took place from September 2021 to January 2022.

The ULPF consisted of three blocks: (i) Block 1 consisted of 31 items containing the food description, food items, and food preparations. The respondents were asked to classify food and preparations as: *in natura*, minimally processed, processed, and ultra-processed. In parallel, the authors created two lists (answers keys), each containing the classification of 31 items according to the established criteria (FGBP and FST). According to FST, the classification was: (i) *in natura*—food of plant or animal origin, for immediate consumption, whose inedible parts are removed; (ii) minimally processed—peeled, cut, washed, packaged, and ready-to-eat fruits and/or vegetables; (iii) processed—food that has been modified from its natural state through processes/operations such as pasteurization, freezing, sterilization, fermentation, food additives, among others [16,17,18,19,20,21]. According to FGBP the groups are classified as: (i) *in natura*—obtained from plants or animals (such as leaves, fruits, eggs, milk) and which do not undergo alteration after leaving nature; (ii) minimally processed—*in natura* food is subject to minimal alterations; (iii) processed—*in natura* food, made with the addition of salt or sugar, or another substance for culinary use; (iv) ultra-processed—obtained entirely from substances extracted from food or synthesized (colorants, flavorings, flavor enhancers, and additives). One whose technique includes extrusion, molding, and pre-processing by frying or cooking [25].

A total of 31 points (100%) was considered for each list to analyze the results. The number of correct answers of each participant for the classification of items was obtained, according to the number of correct answers for the respective lists (FGBP and FST), expressed as a percentage of correct answers. Therefore, it was possible to determine if the participant classified better considering the FGBP or FST parameters; (ii) Block 2 consisted of four items that described the concepts of *in natura*, minimally processed, processed, and ultra-processed. Of these items, three referred to the FST definitions (*in natura*, minimally processed and processed), and four referred to the definitions according to the FGBP (*in natura*, minimally processed, processed, and ultra-processed). For the statistical analysis of Block 2, the respondents who correctly answered all four items referring to the definitions according to the FGBP were defined as “understanders” of the FGBP. As “understanders” of FST, respondents who identified all three items right using the definitions according to FST; (iii) Block 3 contained only one item. Its objective was to identify how the consumer considered it “to be easier” to classify food. Thus, respondents were asked to mark the sub-items by (1) “level of processing”, if this was their answer; (2) food groups, if that was their answer; (3) sources of nutrients, if that was their answer; (4) list of ingredients, if this were his answer to food classification.

In order to apply the ULPF to consumers, sociodemographic data were added, such as nationality, administrative region, the state where they live, age, gender, education level, marital status, persons per residence and monthly income, and theoretical definitions of the food classification considering the Food Guide for the Brazilian Population and food science and technology. According to Hair et al. [26], the validation process of an instrument requires 20 respondents per item (20:1). Therefore, to validate the ULPF, the minimum sample size was estimated at 720 (20:36) participants.

### 2.3. Statistical Analysis

Categorical variables (sociodemographic characteristics) were described as frequencies (n) and percentages (%), and quantitative variables as mean and standard deviation or standard error. Independent Student’s *t*-test, and ANOVA with Tukey’s post hoc tests were used to examine differences in scores. The chi-square test was used to compare categorical variables. The level of statistical significance was set at 5% (*p* < 0.05). The Kuder−Richardson formula 20 (KR-20) calculation was performed to assess the internal consistency and homogeneity since the items required dichotomous responses [27,28]. The statistical software IBM SPSS Statistics for Windows (IBM Corp, Armonk, NY, USA) was used for the analysis.

## 3. Results

### 3.1. “Understanding of the Level of Processing of Food” (ULPF): Construction and Validation

Based on the evaluation and analysis of the food classification adopted in the food-based dietary guidelines (FBDG) of the Food and Agriculture Organization of the United Nations (FAO), 106 items were selected to compose the first version of the ULPF. After evaluation by a panel of experts and interviews, 36 items were approved (Figure 1) to compose the final version of the ULPF, distributed in three (3) blocks (Appendix A, Figure 1). The internal consistency (reliability) of the instrument (and its classifications) was verified using the Kuder−Richardson formula 20 (KR-20) measure. The ULFP showed good internal consistency for the FST classification (KR-20 = 0.672) and the FGBP classification (KR-20 = 0.748) (Figure 1).

### 3.2. “Understanding of the Level of Processing of Food”: Application

Of the initial sample of 2353 participants who accessed the ULPF, 99.1% (n = 2333) agreed to participate in the research by signing the Free and Informed Consent Form and fully answering the ULPF. The nationwide distribution of the participants among the Brazilian regions is presented in Table 1. Participants were mostly from the Southeast Brazilian region (n = 912; 39.1%), followed by the Northeast (n = 567; 24.3%), South (n = 349; 15%), North (n = 268; 11.5%) and Center-west (n = 237; 10.1%). Table 1 shows the methodological rigor of adequacy of 70% or more in the sample representation, according to the last national census [29], since all Brazilian regions achieved this goal.

The majority of the respondents was female (n = 1373; 58.9%). More than half of the respondents (54.4%) were 30–39 years (n = 659; 28.2%) or 40–49 years (n = 611; 26.2%). Most respondents had an educational level equivalent to postgraduation (n = 1682; 72.1%). More than 50% of respondents had an income between 5 and 15 minimum wages (a minimum wage during data collection was 1000,00 BRL, equivalent to 213 USD). More than half of the participants had a partner (n = 1509; 64.7%), and 46.6% (n = 1088) lived with three or more people (Table S1).

Table 2 describes the respondents’ data on food classification as *in natura*, minimally processed, processed, and ultra-processed (Block 1). For each category, those who obtained the three highest scores stood out. Thus, selected, peeled, and cleaned nuts, walnuts, and peanuts (n = 1143; 49%), refrigerated meat (n = 1060; 45.4%), and fruit juice prepared in a restaurant (n = 924; 39.6%) were, more frequently, food classified as *in natura*. As minimally processed food, the highest frequencies were obtained for dried fruits (n = 1193; 51.1%), rice prepared with carrots, green beans, oil, garlic, and salt, packaged or not (n = 1162; 49.8%), and beans cooked at home with water, garlic and salt (n = 1087; 46.6%), while the strawberry cream prepared with corn starch, milk, and sugar (n = 1353; 58%), the curd (n = 1301; 55.8%), plant extracts, such as “almond milk, soy milk, rice milk”, added water and sugar (n = 1293; 55.4%) were classified as processed food (Table 2).

The sociodemographic data associated with the respondents’ responses to the food classification as *in natura*, minimally processed, processed, ultra-processed (Block 2), and raw scores (n = 31 points; 100%), mean, standard deviation, and degree of significance are described in Table 3. According to the FGBP, there was a significant difference (*p* < 0.05) for the variables gender, age group, geographic region, and level of education regarding the food classification. The highest values for the mean score were obtained for females (n = 33.00; SD = 13.79); for the age groups 20–29 years (n = 33.99; SD = 14.33) and 30–39 years (n = 33.28; SD = 13.44); for respondents residing in the South region (n = 34.09; SD = 14.45); and for respondents with an education level equivalent to a graduate degree (n = 32.71; SD = 13.08) (Table 3).

According to the FST, there was a significant difference (*p* < 0.05) for the variables gender, age group, education level, and income regarding the food classification. The highest values for the mean score were obtained for males (n = 42.01; SD = 15.80); for the age group equal to or greater than 60 years (n = 42.04; SD = 17.00), and for respondents with monthly income above 15 minimum wages (n = 43.50; SD = 16.97) (Table 3).

Comparing the results of the scores obtained for Block 1 with the data indicated by the respondents for Block 2, it appears that about 12% (n = 276) of the respondents scored 31 points (Block 1) and answered correctly all Block 2 items, concerning the FGBP classification. Eighty-four (n = 84; 3%) of the respondents scored 31 points (Block 1) and answered correctly all items about the definitions described for the FST (Block 2). Eight-five percent (n = 1973) of the participants did not reach the 31 points in Block 1 and did not answer correctly the items in Block 2, or did not know how to respond to the items in Blocks 1 and 2. Such data suggest that about 85% of the respondents do not understand food classification according to the definitions of the FGBP or FST.

In addition, respondents (n = 276; 12%) with an understanding of the food classification according to the FGBP (Block 1) were the ones who most correctly responded to the items in Block 1 and Block 2 (*p* < 0.05). Respondents (n = 84; 3%) with an understanding of food classification according to FST (Block 1) were the ones who most correctly answered the items in Block 2 (FST) (*p* < 0.05), indicating the small proportion of respondents with an understanding of the food classification, regardless if it was according to FGBP or FST.

Regarding the data from Block 3, whose objective was to identify how the consumer considered “it would be easier” to classify food according to (1) level of processing; (2) food groups; (3) nutrient sources; (4) list of ingredients, it was found that there was a significant difference between the respondents for the variables gender, age, and region.

In general, the results suggest that most respondents understood “it was easier” to classify food according to food groups (n = 1259; 54%), with a significant difference for female respondents (55.6%), for age (40–49 years, n = 55.2% and 50–59 years, n = 57.6%) and respondents living in the Northeast region (57.3%).

Male respondents indicated “it was easier” to classify food according to the source of nutrients (22.3%), as well as respondents 49–49 years (18.2%) and 50–59 years (19.4%) and living in the Center-west region (25.7%). Regarding the classification of food according to the list of ingredients, female respondents (17.3%), up to 20 years (21.7%), and those living in the South region (20.9%) considered this the “easiest” criterion for classifying food.

As for the classification of food according to the level of processing, 11.3% of male respondents, 18.1% of respondents over 60 years, and 13.4% of respondents living in the North region considered this the easiest way to sort food.

## 4. Discussion

This study, with respondents from all Brazilian states, is the first to validate and apply an instrument to assess the consumer’s understanding of the classification of food according to the level of processing, as *in natura* food, minimally processed food, processed food, and ultra-processed food, following the operational definitions of the Food Guide for the Brazilian Population (FGBP) [25] and with the definitions on food processing available in the literature on food science and technology (FST) [16,17,18,19,20,21].

The ULFP validation followed Boateng et al. [12] and the Delphi methodology [13,14,15]. According to Kline [30], the initial set of items developed must be at least twice as large as the desired final scale. The instrument had 106 items in the first stage, 3 times more than the final version, following the recommendation [30].

Regarding the number of experts needed to assess the adequacy of each item on the measurement performed by the ULFP, there is no consensus in the literature on this number [12]. Pasquali [31] considers that six is the minimum number, varying according to the instrument. For other authors, however, this number cannot be too small, as it may prevent the existence of consensus using the application of the Delphi methodology [13,14,15]. The Delphi methodology was adopted because, for the semantic and content analysis, it allows the structure of a group communication process, making it effective and allowing to obtain the total agreement of the items of an instrument when a group of individuals deals with a complex process [12,13,14,15]. The subjective assessment of the ULFP by interviews was also relevant because this technique allows identifying whether the target population’s language used to describe the items is understood [12,22,23,24].

The number of respondents was equal to 2333, showing a significant number of respondents per item according to the recommendation by Hair et al. [26] in the validation process (a minimum number of twenty respondents per item). We also obtained a representative sample from each Brazilian region, with an adequacy of 70%. The KR-20 values were 0.748 for the FGBP classification and 0.672 for the FST classification, indicating that the items assessed the same attribute and produced consistent results. The literature recommends KR-20 values ≥ 0.6 [27,28,32].

In our study, the most significant number of respondents were female (58.9%), as with other surveys, since females tend to care more about health and food and are more available to answer questionnaires [7,8,9,33,34,35,36,37]. Studies assessing consumer food choices have shown that women typically: assume responsibility for managing the household pantry; keeping the family nourished and healthy; being concerned with the feeding of children; being more concerned with health and aspects related to the nutritional value of food, possibly justifying, in such a way, the participation of women in research related to food and nutrition [38,39,40].

Most respondents were between 30 and 49 years old, with a monthly income between 5 and 15 minimum wages. These data agree with the Brazilian Institute of Geography and Statistics (IBGE), which indicates that these are the respondents who, possibly, have more access to the global system of interconnected computer networks (Internet Protocol Suite) [41].

According to the FGBP criteria [25], of the items described in Block 1, 16 are classified as minimally processed food and 15 as ultra-processed food (Table 2). In this sense, according to the FGBP, participants considered minimally processed food as “beans cooked at home, in a restaurant or industry, with water, salt and garlic”, “dried fruits, produced at home, on the farm or in industry”, “rice prepared with carrots, green beans, oil, garlic, and salt, packaged or not”, granola, and rice flour, among others. As ultra-processed food, these participants classified the “breakfast cereal (corn flour, sugar syrup, minerals, salt, vitamins, and food additives, as a flavoring and antioxidant)” and ready-made seasonings (Table 2).

For FGBP, minimally processed food is *in natura* food that has been subjected to cleaning processes, removal of inedible or undesirable parts, fractionation, milling, drying, fermentation, pasteurization, refrigeration, freezing, and similar processes that do not involve aggregation of salt, sugar, oils, fats or other substances to the original food. Ultra-processed foods are industrial formulations made entirely or mainly from substances extracted from food (oils, fats, sugar, starch, proteins), derived from food constituents (hydrogenated fats, modified starch), or synthesized in the laboratory, based on organic materials such as petroleum and charcoal (colorants, flavorings, flavor enhancers and various types of additives used to endow products with attractive sensory properties) [25].

These respondents possibly classified such food according to the conservation method and “level of processing” used, mainly understood when comparing the classification attributed to refrigerated and frozen meat. The first was considered *in natura* food and the second was considered minimally processed by most participants.

What needs to be considered is that “cooking beans and rice” at home, in food service, or in industry, for example, requires a unit operation/a process, called cooking, which uses a binomial time and temperature (heat treatment) specific to each product and sufficient to make it edible, safe and pleasant. Thus, it is prudent to consider that the essential treatments used in industry to transform raw materials into safe and edible products/meals are those used at home and in food catering for food preparation [42].

The application of heat during household cooking of foodstuffs encompasses a variety of processes (boiling, frying, steaming, baking, stewing, roasting, and others) in traditional microwave and steam ovens. Industrial thermal treatment of foodstuffs includes many processes also listed for household cooking (cooking, drying, canning, pasteurization, and related technology (ultra-high temperature treatment—UHT), smoking, and extrusion cooking. However, it is important to note that these processes can be controlled much better on an industrial scale than on a household level. Production volume and equipment size differ between production environments [42,43].

It is important to highlight that cleaning, removal of inedible or undesirable parts, and fractionation are some of the preliminary steps in food processing, regardless of the place of production [42,43,44,45,46,47]. For FST, minimally processed, also called “fresh-cut,” is defined as any fresh fruit or vegetable or any combination that has been physically altered from its original form but remains in a fresh state. Regardless of commodity, it has been trimmed, peeled, washed, and cut into 100% usable product that is subsequently bagged or prepackaged to offer consumers high nutrition, convenience, and value while maintaining freshness. The key point of minimally processed fruit and vegetables is their active metabolism and respiratory rate despite physical changes [17,19,43].

According to Knorr and Watzke [45], minimal processing was developed, especially on-demand from restaurants, catering, and the foodservice industry, to provide pre-cut and pre-prepared vegetable and meat products for meal preparations, saving labor costs and improving hygiene.

Results of studies on the participants’ understanding of the terms used in the FGBP indicate that the food guide does not clearly explain the classification of a particular food in certain groups, such as milk. In this case, both pasteurized milk, ultra-pasteurized milk (long-life/UHT), and dehydrated milk (powder) are classified in the same group—either as *in natura* or as minimally processed food [7,48]. The study by Chagas, Botelho, and Toral [49] showed that participants classified cooked carrot and broccoli-based food as “semi-industrialized” food because they had undergone “minimal processing”. Likewise, these respondents attributed ultra-processed food definitions as industrialized and mega-industrialized food. Industrialized food is food processed through industrial activity [47,50].

Regarding the relationship between the classification and the definitions of food (Blocks 1 and 2), we identified that 85% of the respondents did not understand the classification of food, neither according to the FGBP nor according to FST, even in the case of a sample with a significant number of respondents with high educational level (n = 1682; 72.1%). Furthermore, we also identified that the respondents had an average score of less than 42% for the food classification. The experts in food classification, according to the FGBP, had 10 (32%) of the 31 items correct, while the experts in the food classification of food according to the FST had 13 (42%) right out of the 31 items (Table 3).

In our survey, respondents rated frozen lasagna and ready-to-eat seasonings as ultra-processed food. Menegassi et al. [48] also found that 86% of their survey respondents classified frozen lasagna as ultra-processed food. It is possible to consider the hypothesis that, for the lay consumer, “ready-to-eat food” is an “ultra-processed food” because it was produced according to a formulation that contains numerous ingredients, including food additives, and still has characteristics of convenience food, and practicality (ready to heat/ready to consume) [48,51].

The results of this study are similar to those from the research carried out by Sallaberry et al. [8]. They observed that, although 40% of the participants had more than ten years of completed schooling, they obtained a high percentage of “wrong” answers about the classification proposed by the FGBP, indicating that the respondents did not recognize the terms used to classify food and that such terms are not part of the “daily knowledge” of the studied population [8].

Therefore, part of the consumer misunderstanding about the classification proposed by the food guide stems from the fact that the term ultra-processed is highly confusing [42,43,44,45,46,47,52]. For FGBP, ultra-processed foods are industrial formulations made entirely or mainly from substances extracted from food (oils, fats, sugar, starch, proteins), derived from food constituents (hydrogenated fats, modified starch), or synthesized in a laboratory based on organic materials such as petroleum and coal (colorants, flavorings, flavor enhancers and various types of additives used to endow products with attractive sensory properties) [25].

Health professionals conceived the FGBP, and the proposed classification has been used in studies that associate food and health (nutritional epidemiology) [53,54,55,56,57,58,59]. The jargon “ultra-processed” in public networking has been rapidly spreading, creating immense confusion among consumers and even in the industrial sector, where its interpretation is controversial. The prefix ‘ultra‘ means very, extreme, or radical and may add a good or bad connotation [44,52]. In such a way, it promotes the negative association between the understanding of what is processed food, industrialized food, and food made at home or in food service, for the consumer [44].

It is important to note that no legal standard defines ultra-processed food [52]. Knorr and Augustin [47] contend that an unfortunate oversight uses an existing and well-respected term in food science and technology (i.e., processing) that never had nutritional implications for food classification. The term “process” was initially used to imply a physical, mechanical, or biological manipulation of food or food ingredients. The term ‘‘process” should remain, and appropriate nutritional terminology should be used for any new nutritional classification or status [47]. The use of food processing has enabled the development of safe, nutritious, and sensorially acceptable food. However, it has also led to producing energy-dense food high in fats, salt, and sugars [42,43,44,60,61].

The same food given as ultra-processed food (bread, cakes, frozen lasagna, stroganoff) can be prepared at home or in industrial settings. They have essentially the same attributes, whether culinary preparations or manufactured by the food industry, with the same ingredients and using similar processes. Home-cooked meals will typically have a lengthy list of ingredients yet are not termed ultra-processed food. As a result, the new terminology relating to processed and ultra-processed food can potentially confuse when used for public health messaging [44].

For Carretero et al. [52], rigor is lacking and misleading from a scientific−technical point of view. There is no description of technology but instead of products of varied technologies and compositions and the typology of their ingredients, not their required quality. Science advances by trial and error. Based on this, dietary behavior is defined. In short, it is a concept accepted by some institutions, but it should be revised due to its lack of precision [52].

Processed food is an integral part of the equation for delivering nutrition and food security [42,43,44,60]. Most agricultural raw materials need to be processed for conversion into safe and palatable food and preserved food to enable their distribution through the supply chain. Without food processing for preservation, there will be a breakdown in sustainable food supply chains. The NOVA system can potentially damage the credibility of food preservation operations by applying the term “process” in its classification system.

In our study, the NOVA system was not considered an accurate way to define food. Some of the used classification criteria are ambiguous, inconsistent, and often give less weight to existing scientific evidence on nutrition and food processing effects; critical analysis of these criteria provokes conflict among researchers [61]. In this way, according to Petrus et al. [44], consumers need to be correctly informed that healthiness has no direct or absolute correlation either with the number of ingredients, intensity, or several processes or with the fact that the food has been processed in households or a large industrial plant [44].

Food-based dietary guidelines are globally recognized as tools for food and nutrition education. Their fundamental objective is to promote the health of populations through a set of recommendations for choosing, preparing, and consuming food. More than one hundred countries, in line with the guidance of the United Nations Food and Agriculture Organization, already have such a document based on the eating habits of their people [1,2,3,4,6,62,63,64]. In this way, the consumer’s understanding of the classification and composition of food is crucial for establishing their food choices and developing studies on nutritional epidemiology. For the consumer, some information is often technical and not always understandable, even with the advances arising from mandatory nutrition labeling for processed food [65,66,67].

Thus, the food classification in food-based dietary guidelines must follow some principles: contain a classification system understandable by the target population; the consumption of food and eating habits of the country to which it is intended, and the use of recommendations for the most prevalent and incident diseases in each country [2,4,5,6,62,63].

Some research carried out to identify Brazilian consumers’ knowledge about FGBP had different objectives and scopes from the present study. Menegassi et al. [48] conducted an intervention study with 72 participants to evaluate their knowledge about food classification according to the food guide. The results obtained by these authors showed that only after the intervention (minicourse) did the participants have a better understanding of the classification, considering the highest score obtained for the correct answers [48].

Research conducted by Menegassi et al. [7] evaluated the participants’ knowledge (n = 69), with students entering and concluding a nutrition course about the food classification described in the FGBP. According to the food classification mentioned above, the authors obtained similar results regarding the number of correct answers [7]. The study of Chagas, Botelho, and Toral [49] qualitatively evaluate adolescents’ understanding of FGBP messages (n = 141). At the same time, research by Figueiredo [9] aimed to identify the knowledge of professionals (n = 25) in the health area and whether these professionals applied the FGBP in their work. Both obtained similar results. Other studies carried out outside Brazil also assessed consumer knowledge and/or understanding of the “level of food processing” using the terms “ultra-processed food” and the term “food processing”. Ares et al. [51] analyzed how 2381 Uruguayans conceptualized ultra-processed food and concluded that only 8.8% (n = 210) of the participants could not understand the meaning of the term ultra-processed food. However, some participants perceived industrialized food, culinary ingredients, and even some minimally processed food as ultra-processed [51].

A study by Aguirre et al. [67] qualitatively evaluated how 181 university students, Ecuadorians, and Argentines, answered the question: What do you understand by ultra-processed food? Moreover, it asked how they would classify ultra-processed food. The results showed that participants understand ultra-processed food as highly processed food containing various artificial ingredients. Like Ares et al. [51], food such as meat and milk were wrongly understood as processed food.

Using the focus group technique, Bleiweiss-Sande et al. [68] surveyed 53 North American children (9–12 years old) to assess how they interpreted terms related to food processing. The results showed that the children had an understanding of food processing. However, the children did not show agreement in listing highly processed food. From a methodological point of view, these studies had main weakness because their questionnaires were not validated for the target audience [68].

Understanding that man is omnivorous and, thus, his food choices can be influenced by different conditions, such as cultural, economic, social, religious, biological, and sensory [38], participants were asked to indicate which would be the “easiest” system to understand food classification by level of processing; food groups; nutrient sources, or ingredient list. Regarding classifying food more efficiently (Block 3), the data obtained in this survey suggest that most respondents believed it was easier to classify food according to food groups (n = 1259;54%). This result agrees with the food classification presented in food-based dietary guidelines [6]. At the same time, only 10.5% (n = 245) of respondents believed it was easier to classify food by level of processing. Only 16.3% (n = 380) of respondents believed it was easier to classify food according to the ingredients list.

According to Botelho, Araújo, and Pinelli [43], more than the classification of food, it is important to evaluate the chemical and nutritional composition of simple and compound food. Recipes, technical preparation files, and formulations consist of instructions on the quantity and quality of raw materials and ingredients, accurate recording of all ingredients, proportions, and sequence of operations. Systematized recipes, technical preparation files, and formulations reveal food’s chemical and nutritional composition and show trends in the relationship between food and nutrition [43].

Thus, the need to harmonize the nomenclature used to classify food becomes evident, especially in food guides, due to its role in disseminating information to the population [2,6,43,62,65,66,69]. Furthermore, through the data listed in food guides, many surveys on food consumption are developed to assess a group’s nutritional status.

It is an urgent requirement to expand the interdisciplinarity of the areas involved and the dialogue to develop a rational scientific approach regarding the importance of the industrialization of food and nutrition as a fundamental factor in the consumer’s quality of life. Moreover, it transforms data on nutrition, food science, and technology into evidence-based public/official messages for the population.

Some limitations should be mentioned, such as the use of an online survey in which the sample was collected through non-random probability, making it difficult to generalize the results. However, due to the COVID-19 pandemic, we decided to disseminate the survey online. It was less costly and less invasive, requiring less effort and time for the researchers and participants than a face-to-face interview since Brazil is a continent-wide country. Despite the study’s strength, with a large sample of respondents, the internet spread could be a potential limitation because of the difficulty of accessing the questionnaire by individuals who do not have access to the internet. This may have contributed to greater participation of individuals with greater access to the internet, as discussed, with a higher level of education and income. However, considering the objective of covering all Brazilian states, disseminating the questionnaire through the internet was the most economical and feasible for the Brazilian reality.

It is relevant for the consumer to understand food classification and nutritional values. Further studies could be performed with a probabilistic random sample, making it possible to obtain greater results on consumer understanding in different social strata and age groups. Our data will be helpful in the development of a proposal for classifying food based on the nutritional value of the food or preparations or industrialized products. Additionally, the information will be important to consolidate information contained in food guides and literature on FST for the consumer.

## 5. Conclusions

This study successfully developed and validated an instrument to evaluate consumers’ understanding of food processing. The participants showed that it was difficult to understand food classifications according to the level of processing. About 85% of them did not understand, did not know, or did not know how to define or classify food according to the criteria adopted in this study (FST and FGBP). Likewise, consumers did not show a logical understanding of classifying food according to the level of processing proposed by the FGBP and FST.

More than 50% of the participants believed that it was easier to understand food classification according to food groups (cereals; legumes; meat and derivatives; milk and dairy products; fruits and vegetables; oils and fats; and sugars), following the guidelines described in the first food guides, from the middle of the last century. Therefore, why should food guides and food classification systems be based on classifications and nomenclatures that are not understandable or that are difficult for the population to access cognitively?

This study’s results point to the need for more studies aimed at different population groups to identify how easily the population understands food classification and how it should be proposed, especially for FGBP. Food guides are instruments of nutrition education committed to reaching the entire population (regardless of their biological, social, cultural, or economic condition). They also indicate the urgent approach between public policymakers and FST professionals so that they can create effective systems based on scientific knowledge to systematize food, ingredients, and food preparation classifications. Furthermore, the dissemination of scientific knowledge on social media is recommended as a strategy to widely disseminate the correct use of information and contribute to the population’s food choices.

## Figures and Tables

**Table 1:** National distribution of participants.

Region	Brazilian Population		Participants		
	(n)	(%)	(n)	(%)	Adequacy
Center-west	16,297,074	7.75	237	10.1	130%
Northeast	57,071,654	27.15	567	24.3	89%
North	18,430,980	8.77	268	11.5	131%
Southeast	88,371,433	42.05	912	39.1	93%
South	29,975,984	14.26	349	15	105%
OVERALL	210,147,125	100%	2333	100%	All in Accordance

**Table 2:** Number and frequency of respondents for the general classification of food.

Items	*In Natura*		Minimally Processed		Processed		Ultra-Processed		I Do Not Know		Classification	
	(n)	(%)	(n)	(%)	(n)	(%)	(n)	(%)	(n)	(%)	FGBP *	FST *
Refrigerated meat is:	1060	45.4	923	39.6	217	9.3	14	0.6	119	5.1	Minimally processed	*In natura*
“Feijoada” prepared at home with black beans, pork meat, sausage, laurel, pepper, and salt is:	185	7.9	828	35.5	971	41.6	263	11.3	86	3.7	Ultra-processed	Processed
Parboiled rice is:	171	7.3	931	39.9	972	41.7	105	4.5	154	6.6	Minimally processed	Processed
Pasteurized milk is:	113	4.8	872	37.4	1034	44.3	270	11.6	44	1.9	Minimally processed	Processed
Homemade stroganoff with meat, sour cream, ketchup, Worcestershire sauce, mushrooms, olive oil, salt, pepper, and brandy is:	79	3.4	457	19.6	1130	48.4	590	25.3	77	3.3	Ultra-processed	Processed
Beans cooked at home, in a restaurant, or in industry, with water, salt and garlic are:	747	32	1087	46.6	435	18.6	9	0.4	55	2.4	Minimally processed	Processed
Granola containing various cereal flakes, palm fat, grated coconut, brown sugar, honey, raisins, and Brazil nuts is:	374	16	997	42.7	791	33.9	119	5.1	52	2.2	Ultra-processed	Processed
Frozen meat is:	676	29	1032	44.2	477	20.4	66	2.8	82	3.5	Minimally processed	*In natura*
Natural yogurt is:	376	16.1	984	42.2	1275	54.7	663	28.4	42	1.8	Minimally processed	Processed
Hot dog bread prepared with wheat flour, sugar, yeast, vegetable fat, salt, soy flour, and preservatives is:	38	1.6	310	13.3	1275	54.7	663	28.4	47	2	Ultra-processed	Processed
Dried fruits, produced at home, on the farm, or in industry are:	601	25.8	1193	51.1	466	20	29	1.2	44	1.9	Minimally processed	Processed
Peanut butter with concentrated vegetable protein, cocoa, and sugar is:	60	2.6	392	16.8	1256	53.8	565	24.2	60	2.6	Ultra-processed	Processed
Powder to prepare coffee is:	188	8.1	1030	44.1	1008	43.2	73	3.1	34	1.5	Minimally processed	Processed
Curd containing sugar, milk yeast, and gelatin is:	83	3.6	601	25.8	1301	55.8	280	12	68	2.9	Ultra-processed	Processed
Fruit juice prepared in a restaurant is:	924	39.6	970	41.6	325	13.9	38	1.6	76	3.3	Minimally processed	Processed
Rice flour is:	200	8.6	1059	45.4	915	39.2	62	2.7	97	4.2	Minimally processed	Processed
Refreshment and fruit nectar are:	159	6.8	320	13.7	998	42.8	798	34.2	58	2.5	Ultra-processed	Processed
Frozen lasagna (lasagna noodles, chicken, mozzarella cheese, oil, tomato sauce, green seasoning, salt, and chicken broth) is:	19	0.8	115	4.9	839	36	1320	56.6	40	1.7	Ultra-processed	Processed
Plant extracts such as “almond milk, soy milk, and rice milk”, added with water and sugar are:	78	3.3	499	21.4	1293	55.4	31	16.8	72	3.1	Ultra-processed	Processed
Sweet “Canjica” prepared with milk, sugar, condensed milk, coconut milk, and grated coconut, is:	90	3.9	603	25.8	1281	54.9	292	12.5	67	2.9	Ultra-processed	Processed
Chester^®^, containing salt, glucose, and food additives, ready for heating and consumption is:	19	0.8	107	4.6	752	32.2	1398	59.9	57	2.4	Ultra-processed	Processed
Seasonings, containing salt, starch, vegetable fat, sugar, parsley, garlic, flavoring and coloring, are:	27	1.2	147	6.3	727	31.2	1393	59.7	39	1.7	Minimally processed	Processed
Instant whole milk powder is:	25	1.1	312	13.4	1252	53.7	704	30.2	40	1.7	Minimally processed	Processed
Chestnuts, walnuts, and peanuts selected, peeled, and cleaned are:	1143	49	1001	42.9	149	6.4	12	0.5	28	1.2	Minimally processed	Processed
Strawberry cream, prepared with corn starch (Maizena^®^), milk, and sugar, is:	48	2.1	584	25	1353	58	279	12	69	3	Ultra-processed	Processed
Rice prepared with carrots, green beans, oil, garlic, and salt, packed or not, is:	317	13.6	1162	49.8	738	31.6	49	2.1	67	2.9	Minimally processed	Processed
Hamburger with minced meat and salt is:	228	9.8	726	31.1	937	40.2	395	16.9	47	2	Ultra-processed	Processed
Cake, prepared with corn flour, sugar, corn, eggs, coconut milk, and chemical yeast, is:	59	2.5	620	26.6	1332	57.1	275	11,8	47	2	Ultra-processed	Processed
Cereal, containing corn flour, sugar syrup, minerals, salt, vitamins, and food additives such as flavoring and antioxidants, is:	24	1	149	6.4	802	34.4	1306	56	52	2.2	Ultra-processed	Processed
Pasta, as spaghetti, based on wheat flour and water, is:	100	4.3	798	34.2	1277	54.7	114	4.9	44	1.9	Minimally processed	Processed
Pre-cooked rice, based on brown rice, with dehydrated vegetables (corn, peas, broccoli, and carrots), packaged, is:	100	4.3	728	31.2	1152	49.4	292	12.5	61	2.6	Minimally processed	Processed

* Food Guide for the Brazilian Population (FGBP) and food science and technology (FST).

**Table 3:** Relationship between the mean score (%) and the standard deviation for respondents who classified food according to the definitions of the Food Guide for the Brazilian Population (FGBP) and the definitions of food classification according to food science and technology (FST).

Characteristics	Classification	Category	Mean Score	Standard Deviation	*p*-Value
Gender	FGBP	Male	30.63	12.66	*p* = 0.00
		Female	33.00	13.79	
	FST	Male	42.01	15.80	*p* = 0.00
		Female	39.57	16.59	
Age	FGBP	Up to 20	28.29	12.64	*p* = 0.00
		20–29	33.99	14.33	
		30–39	33.28	13.44	
		40–49	31.89	13.35	
		50–59	31.12	13.16	
		Over 60	29.15	11.74	
	FGBP	Up to 20	33.41	15.73	*p* = 0.00
		20–29	38.38	16.15	
		30–39	41.47	16.57	
		40–49	40.76	15.51	
		50–59	41.38	16.43	
		Over 60	42.04	17.00	
Marital status	FGBP	With partner	32.00	13.33	*p* = 0.925
		Without partner	32.06	13.49	
	FST	With partner	41.14	16.23	*p* = 0.025
		Without partner	39.55	16.42	
Region	FGBP	Center-west	28.96	13.59	*p* = 0.00
		Northeast	31.74	12.77	
		North	30.33	12.84	
		Southeast	32.70	13.26	
		South	34.09	14.45	
	FST	Center-west	40.24	16.72	*p* = 0.476
		Northeast	39.60	15.59	
		North	40.76	15.35	
		Southeast	41.20	16.83	
		South	40.61	16.53	
Educational level	FGBP	Up to high school	29.99	14.16	*p* = 0.00
		College degree	30.69	13.72	
		Postgraduation	32.71	13.08	
	FST	Up to high school	35.83	15.86	*p* = 0.00
		College degree	39.93	16.69	
		Postgraduation	41.82	16.16	
Number of people per residence	FGBP	2	32.58	13.13	*p* = 0.191
		3 or more	31.51	13.62	
		1	32.10	13.28	
	FST	2	40.58	16.03	*p* = 0.170
		3 or more	40.15	16.49	
		1	42.21	16.53	
Income—Minimum wage—MW (BRL 1000,00) 1 USD = BRL 5.16	FGBP	Up to 4 MW	31.26	14.25	*p* = 0.064
		From 5 to 9 MW	33.11	13.15	
		From 10 to 15 MW	31.69	12.20	
		Above 15 MW	32.45	13.60	
	FST	Up to 4 MW	38.28	16.70	*p* = 0.00
		From 5 to 9 MW	40.37	14.89	
		From 10 to 15 MW	42.47	16.34	
		Above 15 MW	43.50	16.97	

## Data Availability

Not applicable.

## Supplementary Materials

The following supporting information can be downloaded at: https://www.mdpi.com/article/10.3390/foods11162396/s1, Table S1: Sociodemographic data of Brazilian consumers participating in the ULPF research, 2022.

## Appendix A. Understanding of the “Level of Processing” of Food (ULPF)—Application

**BLOCK 1**

Indicate, in your opinion, the “level of processing” for the following items—FOOD/PREPARATIONS/FOOD PRODUCTS.

Refrigerated meat is: *In natura* Minimally processed Processed Ultra-processed I do not know
“Feijoada” prepared at home with black beans, pork meat, sausage, laurel, pepper, and salt is: *In natura* Minimally processed Processed Ultra-processed I do not know
Parboiled rice is: *In natura* Minimally processed Processed Ultra-processed I do not know
Pasteurized milk is: *In natura* Minimally processed Processed Ultra-processed I do not know
Homemade stroganoff with meat, sour cream, ketchup, Worcestershire sauce, mushrooms, olive oil, salt, pepper, and brandy is: *In natura * Minimally processed Processed Ultra-processed I do not know
Beans cooked at home, in a restaurant, or industry, with water, salt and garlic are: *In natura * Minimally processed Processed Ultra-processed I do not know
Granola containing various cereal flakes, palm fat, grated coconut, brown sugar, honey, raisins, and Brazil nuts is: *In natura * Minimally processed Processed Ultra-processed I do not know
Frozen meat is: *In natura * Minimally processed Processed Ultra-processed I do not know
Natural yogurt is: *In natura * Minimally processed Processed Ultra-processed I do not know
Hot dog bread prepared with wheat flour, sugar, yeast, vegetable fat, salt, soy flour, and preservatives is: *In natura * Minimally processed Processed Ultra-processed I do not know
Dried fruits, produced at home, on the farm, or in industry are: *In natura * Minimally processed Processed Ultra-processed I do not know
Peanut butter with concentrated vegetable protein, cocoa, and sugar is: *In natura * Minimally processed Processed Ultra-processed I do not know
Powder to prepare coffee is: *In natura * Minimally processed Processed Ultra-processed I do not know
Curd containing sugar, milk yeast, and gelatin is: *In natura * Minimally processed Processed Ultra-processed I do not know
Fruit juice prepared in a restaurant is: *In natura * Minimally processed Processed Ultra-processed I do not know
Rice flour is: *In natura * Minimally processed Processed Ultra-processed I do not know
Refreshments such as fruit nectar are: *In natura * Minimally processed Processed Ultra-processed I do not know
Frozen lasagna (lasagna noodles, chicken, mozzarella cheese, oil, tomato sauce, green seasoning, salt, and chicken broth) is: *In natura * Minimally processed Processed Ultra-processed I do not know
Plant extracts such as “almond milk, soy milk, and rice milk,” with added water and sugar are: *In natura * Minimally processed Processed Ultra-processed I do not know
Sweet “Canjica” prepared with milk, sugar, condensed milk, coconut milk, and grated coconut, is: *In natura * Minimally processed Processed Ultra-processed I do not know
Chester^®^, containing salt, glucose, food additives, ready for heating and consumption is: *In natura * Minimally processed Processed Ultra-processed I do not know
Seasonings, containing salt, starch, vegetable fat, sugar, parsley, garlic, flavoring, and coloring, are: *In natura * Minimally processed Processed Ultra-processed I do not know
Instant whole milk powder is: *In natura * Minimally processed Processed Ultra-processed I do not know
Chestnuts, walnuts, and peanuts selected, peeled, and cleaned are: *In natura * Minimally processed Processed Ultra-processed I do not know
Strawberry cream, prepared with corn starch (Maizena^®^), milk, and sugar, is: *In natura * Minimally processed Processed Ultra-processed I do not know
Rice prepared with carrots, green beans, oil, garlic, and salt, packed or not, is: *In natura * Minimally processed Processed Ultra-processed I do not know
Hamburger with minced meat and salt is: *In natura * Minimally processed Processed Ultra-processed I do not know
Cake, prepared with corn flour, sugar, corn, eggs, coconut milk, and chemical yeast, is: *In natura * Minimally processed Processed Ultra-processed I do not know
Cereal, containing corn flour, sugar syrup, minerals, salt, vitamins, and food additives such as flavoring and antioxidants, is: *In natura * Minimally processed Processed Ultra-processed I do not know
Pasta, as spaghetti, based on wheat flour and water, is: *In natura * Minimally processed Processed Ultra-processed I do not know
Pre-cooked rice, based on brown rice, with dehydrated vegetables (corn, peas, broccoli, and carrots), packaged, is: *In natura * Minimally processed Processed Ultra-processed I do not know

**BLOCK 2**

Indicate, according to your opinion, what *in natura*, minimally processed, processed, and ultra-processed foods.

For you *in natura* foods are: (a) Obtained from plants or animals (such as leaves, fruits, eggs, milk) and which do not undergo alteration after leaving nature. (b) Food of plant or animal origin, whose inedible parts are removed for immediate consumption. (c) I do not know.
For you minimally processed food is: (a) *In natura* food is subject to minimal alterations. (b) Peeled, cut, washed, packaged, and ready-to-eat fruits and/or vegetables. (c) I do not know.
For you processed food is: (a) *In natura* food, made with the addition of salt or sugar, or another substance for culinary use. (b) Food that has been modified from its natural state through processes/operations such as pasteurization, freezing, sterilization, fermentation, food additives, among others. (c) I do not know.
For you ultra-processed food is: (a) That obtained entirely from substances extracted from food or synthesized (colorants, flavorings, flavor enhancers, and additives). (b) One whose technique includes extrusion, molding, and pre-processing by frying or cooking. (c) I do not know.

**BLOCK 3**

Indicate how you find it easier to choose foods.

**How easy is it for you to choose foods:**
(a) “Processing level”. Examples: *in natura*, minimally processed, processed, and ultra-processed. (b) Food groups. Examples: grains, meat, fruits and vegetables, and milk and eggs. (c) Nutrient sources. Examples: carbohydrate source, protein source, fat source, vitamin source, mineral source. (d) Ingredients list—the main ingredient, followed by the others (descending order).
