# Supplementary material for: How Do Consumers Understand Food Processing? A Study on the Brazilian Population
Source: Foods. 2022 Aug 10;11(16):2396. doi: 10.3390/foods11162396 (PMC9407463; doi:10.3390/foods11162396)
Supplement: Supplementary file 1 [file foods-11-02396-s001.zip › foods-1805003 - supplementary.pdf]

Table S1: Sociodemographic data of Brazilian consumers participating in the ULPF research, 2022.

**Characteristics**	**Category**	**Respondents**	
		N	Percentage
Gender	Male	960	41.1
	Female	1.373	58.9
Age (Years)	Up to 20	92	3.9
	20 -29	326	14
	30-39	659	28.2
	40-49	611	26.2
	50-59	408	17.5
	Over 60	237	10.2
Marital status	With partner	1509	64.7
	Without partner	824	35.3
Educational level	Up to High School	408	17.5
	College degree	243	10.4
	Post-graduation	1682	72.1
Number of people per residence	1	281	12.1
	2	964	41.3
	3 or more	1088	46.6
Income – Minimum wage (R$1,100.00) 1 US$ = R$ 5.16	Up to 4	695	29.8
	From 5 to 9	648	27.8
	from 10 to 15	625	26.8
	Above 15	242	10.3
	Not informed	123	5.3
